# Correction: Genetic interactions among ADAMTS metalloproteases and basement membrane molecules in cell migration in *Caenorhabditis elegans*

**DOI:** 10.1371/journal.pone.0261880

**Published:** 2021-12-21

**Authors:** Ayaka Imanishi, Yuma Aoki, Masaki Kakehi, Shunsuke Mori, Tomomi Takano, Yukihiko Kubota, Hon-Song Kim, Yukimasa Shibata, Kiyoji Nishiwaki

## Notice of Republication

This article was republished on January 25, 2021, to correct errors in the symbols. In the originally published version, the Greek “alpha” symbol (α) appeared incorrectly as either the letter “a” or as a corrupted symbol. Please download this article again to view the correct version. The originally published, uncorrected article and the republished, corrected articles are provided here for reference.

## Supporting information

S1 File Originally published, uncorrected article. (PDF)

S2 File Republished, corrected article. (PDF)
