# Supplementary figures and images for: Genetic interactions among ADAMTS metalloproteases and basement membrane molecules in cell migration in Caenorhabditis elegans
Source: PLoS One. 2020 Dec 2;15(12):e0240571. doi: 10.1371/journal.pone.0240571 (PMC7710118; doi:10.1371/journal.pone.0240571)

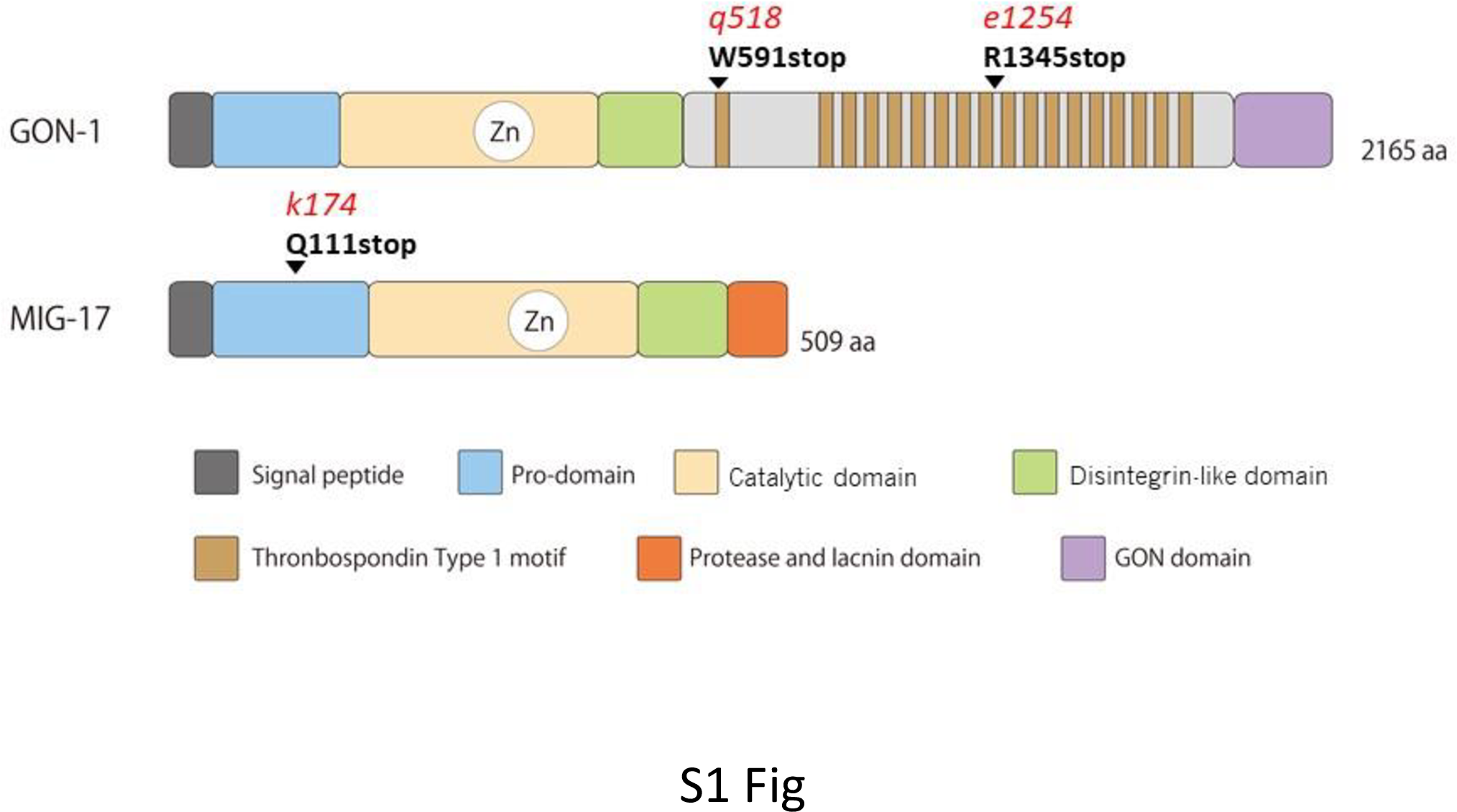

Supplement: S1 Fig — Domain organization is shown by colored boxes. Mutation sites for gon-1(q518 and e1254) and mig-17(k174) are indicated. (TIF) [file pone.0240571.s001.tif]

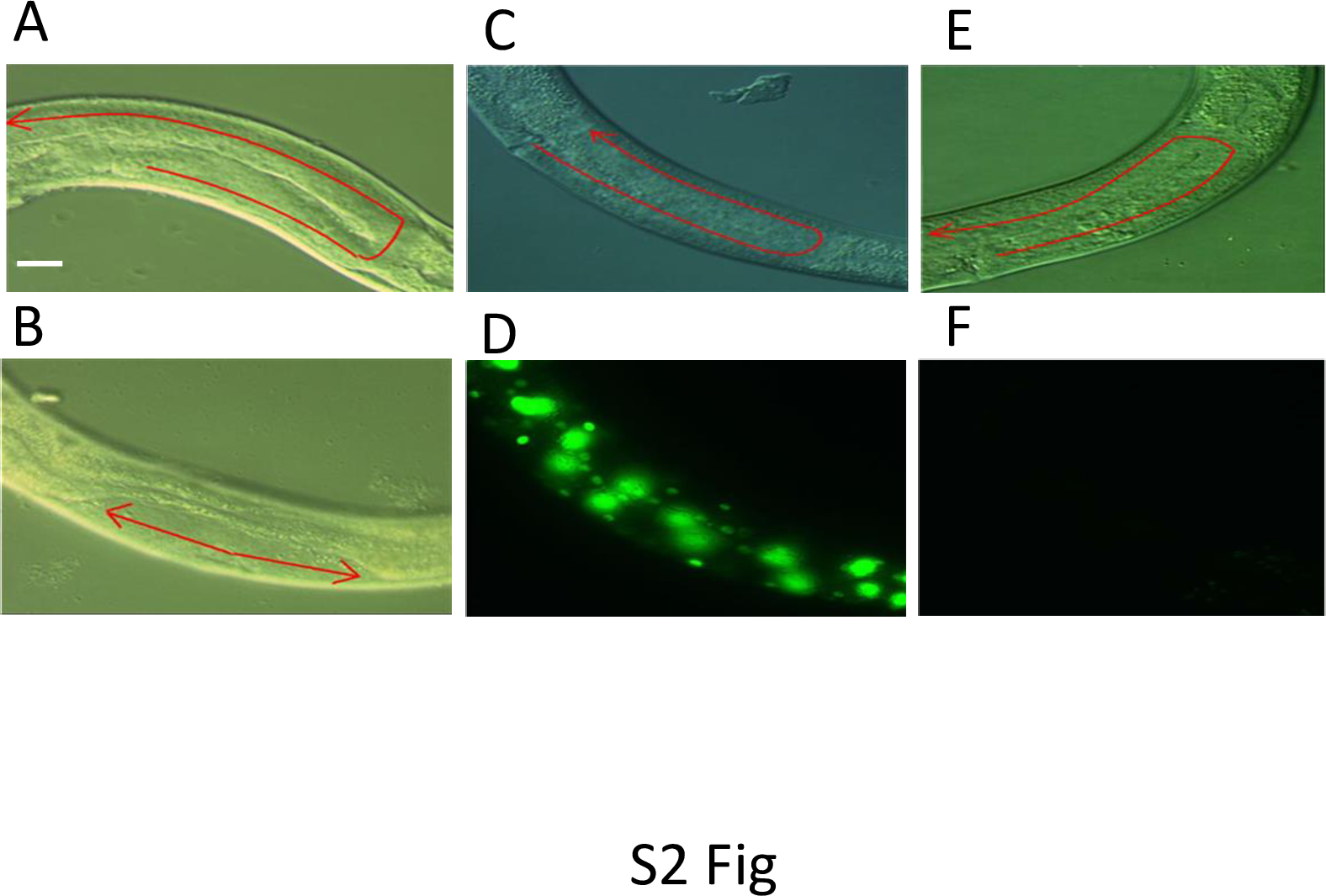

Supplement: S2 Fig — Gonad arms (arrows) of young adult hermaphrodites are shown. A, B. Nomarski images of wild type (A) and gon-1(e1254) (B). C, D. Nomarski (C) and fluorescence (D) images of a gon-1(e1254); tkEx370[gon-1 fosmid, rol-6(su1006), sur-5::GFP] young adult hermaphrodite. E, F. Nomarski (E) and fluorescence (F) images of a let-2(k101); gon-1(e1254) young adult hermaphrodite, which lost the tkEx370[gon-1 fosmid, rol-6(su1006), sur-5::GFP] extrachromosomal array. Bar: 20 μm. (TIF) [file pone.0240571.s002.tif]

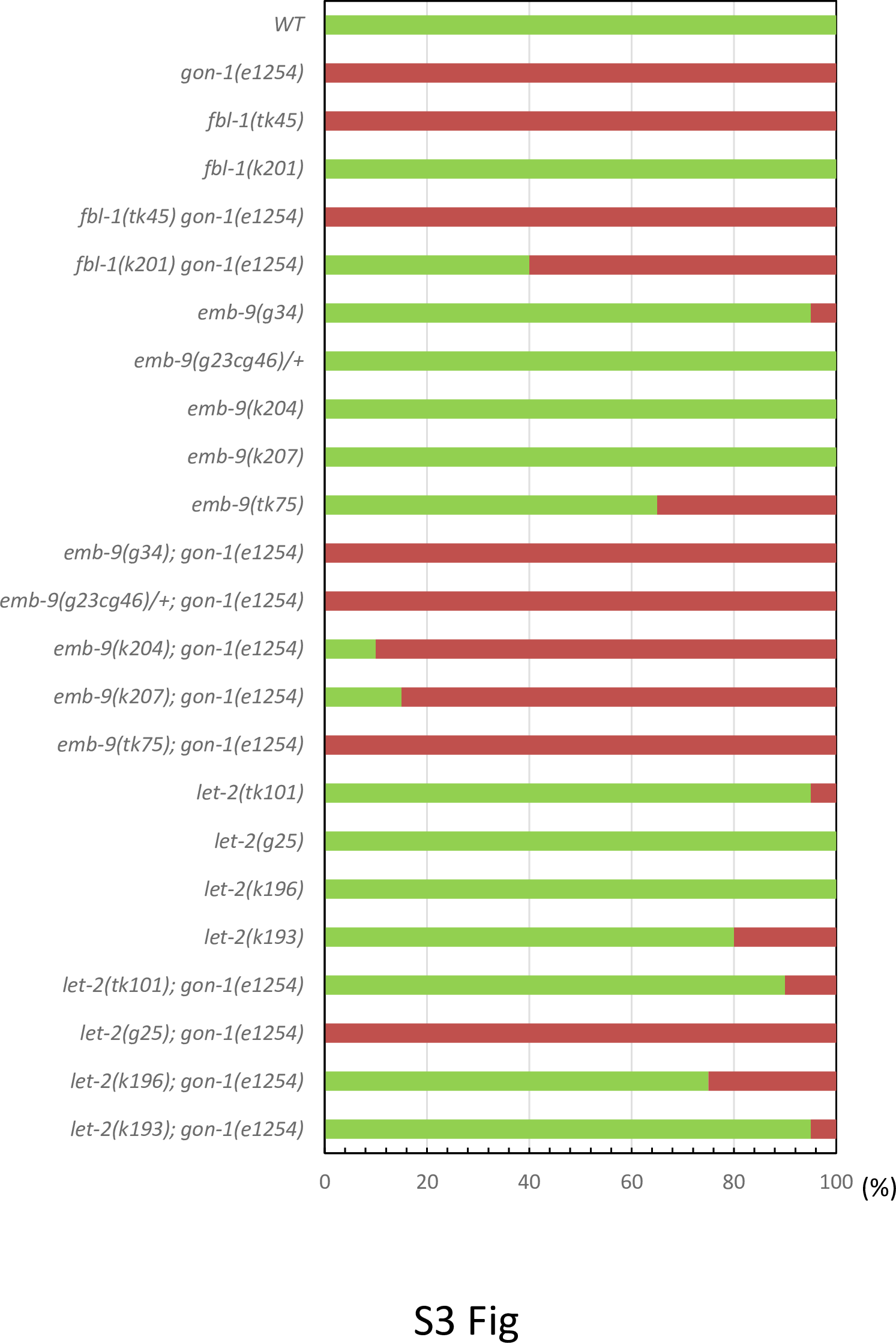

Supplement: S3 Fig — For each strain, 20 first larval stage hermaphrodites were grown at 20°C, and the number of animals that produced offspring were counted. Green and red bars represent % fertile and % sterile animals, respectively. (TIF) [file pone.0240571.s003.tif]
